# A dataset of neonatal EEG recordings with seizure annotations

**DOI:** 10.1038/sdata.2019.39

**Published:** 2019-03-05

**Authors:** N. J. Stevenson, K. Tapani, L. Lauronen, S. Vanhatalo

**Affiliations:** 1 BABA Center, Children’s Hospital, HUS Medical Imaging Center, Department of Clinical Neurophysiology, Helsinki University Hospital, Helsinki, Finland; 2 Clinicum, University of Helsinki, Helsinki, Finland; 3 Brain Modelling Group, QIMR Berghofer Medical Research Institute, Brisbane, Australia

**Keywords:** Brain imaging, Neonatal brain damage, Diagnostic markers

## Abstract

Neonatal seizures are a common emergency in the neonatal intensive care unit (NICU). There are many questions yet to be answered regarding the temporal/spatial characteristics of seizures from different pathologies, response to medication, effects on neurodevelopment and optimal detection. The dataset presented in this descriptor contains EEG recordings from human neonates, the visual interpretation of the EEG by the human experts, supporting clinical data and codes to assist access. Multi-channel EEG was recorded from 79 term neonates admitted to the NICU at the Helsinki University Hospital. The median recording duration was 74 min (IQR: 64 to 96 min). The presence of seizures in the EEGs was annotated independently by three experts. An average of 460 seizures were annotated per expert in the dataset; 39 neonates had seizures and 22 were seizure free, by consensus. The dataset can be used as a reference set of neonatal seizures, in studies of inter-observer agreement and for the development of automated methods of seizure detection and other EEG analyses.

## Background & Summary

Neonatal seizures are regularly seen in infants in the neonatal intensive care unit (NICU). While there is a lack of evidence on the causal link between neonatal seizures and neurodevelopmental outcome in a range of aetiologies in the human neonate, they are aggressively treated with a sequence of anti-epileptic medications^1–4^. Current treatment aims to minimise the seizure burden, i.e. the accumulated duration of seizures^5^.

The identification of seizures in neonates is not trivial^5,6^. Clinical recognition is based on observing stereotypical movements or other ictal signs, which is confounded by a myriad of comparable non-epileptic behaviours^7^. The international gold standard of neonatal seizure diagnosis is, therefore, based on finding specific electrographic discharges in the multi-channel electroencephalogram (EEG)^8,9^. This ultimate solution is, however, challenged by the poor availability of expertise for EEG review in the NICU where immediate identification of seizures is required. A widely adopted bridging solution is to use a time- and amplitude-compressed display of a limited number of EEG electrodes, known as amplitude-integrated EEG^10^. While aEEG is simpler to interpret and improves cot-side seizure detection, it is still subjective, requires special expertise, and is less reliable than the interpretation of the multi-channel EEG by the human expert^11^.

A practical cot-side solution for the current stalemate in neonatal seizure diagnostics, or newborn brain monitoring in general, is automated methods of seizure detection^12^. Ideally, these methods should generate an EEG interpretation that is indistinguishable from the human expert. There have been many attempts to develop neonatal seizure detection algorithms^13–19^. The outputs of these methods have not, however, achieved the benchmark of inter-observer agreement between human experts. Further development of these algorithms is stymied by 1) a lack of openly accessible datasets to evaluate and compare competing algorithms and 2) insufficient methods of assessing algorithm performance due to methodological flaws that do not take into account inter-observer variability of the EEG annotations (e.g. the use of sensitivity and specificity as metrics or a lack of annotations from multiple experts).

This data descriptor outlines a dataset of multi-channel EEG recordings that have been annotated for neonatal seizures by three independent experts. It was compiled for our recent study on neonatal seizure detection algorithms^19^ with parts of the dataset used in another study on inter-observer agreement of neonatal EEG seizure detection^20^. Our dataset can be used as training data for neonatal seizure detection algorithms or as a validation set to compare existing methods. It is the only publicly available dataset of neonatal EEG recordings with annotations of seizures from multiple experts. There have been several EEG datasets containing seizures from older age groups but these datasets either do not have sufficient amounts of neonatal EEG, or a clinically relevant EEG annotation (University of Freiburg; http://epilepsy.uni-freiburg.de/freiburg-seizure-prediction-project/eeg-database, CHB-MIT Database; https://www.physionet.org/pn6/chbmit/). In addition, our present dataset is also useful as a reference dataset for various studies on normal and abnormal EEG patterns commonly seen in the NICU, as well as for studies of inter-observer agreement in visual EEG interpretation.

## Methods

These methods are expanded versions of descriptions in our related work^19,20^.

### Cohort

EEGs were selected from an archive of neonatal EEG recordings that were recorded on request from the clinical team due to suspicion of seizures. All recordings were performed at the Helsinki University Hospital between 2010 and 2014. The majority of infants were admitted to the NICU. Neonates were excluded from this dataset if they were extremely or very preterm (less than 32 weeks gestational age), had a post-menstrual age (PMA) at the time of recording less than 35 or greater than 45 weeks, if there was insufficient supporting clinical data, or if the technical quality of the EEG recording was poor. The lower limit of gestational age exceeds traditional definitions of normal gestation and, in this case, takes into account both variability in the period of gestation and EEG characteristics of maturation^21,22^. A total of 87 neonates were initially included in the cohort. Eight neonates were excluded resulting in a cohort of EEG recordings from 79 neonates.

### EEG Recording

**Figure 1:**
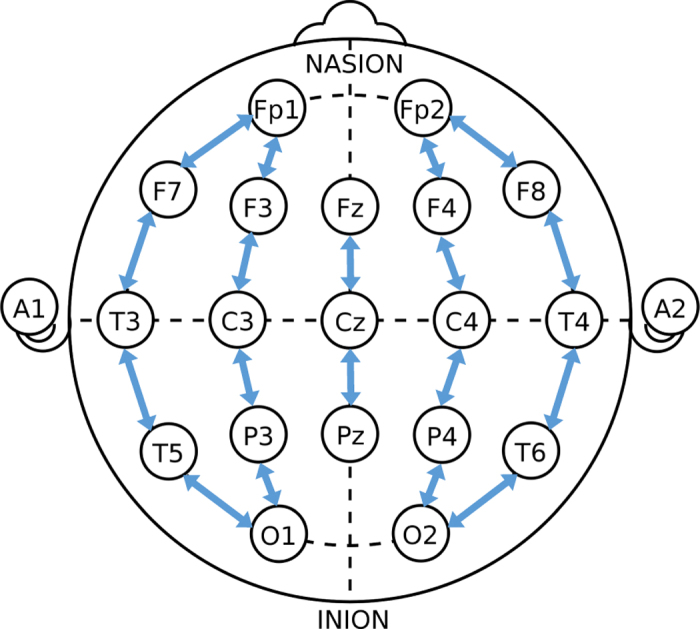
The bipolar EEG montage used by reviewers to annotate the presence of seizures.

Each EEG recording lasted approximately one hour; median recording duration was 74 mins (IQR: 64 to 96 mins). The EEG signals were recorded with a NicOne EEG amplifier (sampling frequency of 256 Hz; Natus, USA) and EEG caps (sintered Ag/AgCl electrodes; Waveguard, ANT-Neuro, Germany) with 19 electrodes positioned as per the international 10–20 standard, including a recording reference at midline. A bipolar montage was generated for annotation according to the standard longitudinal bipolar layout (a.k.a. ‘double banana’): Fp2-F4, F4-C4, C4-P4, P4-O2, Fp1-F3, F3-C3, C3-P3, P3-O1, Fp2-F8, F8-T4, T4-T6, T6-O2, Fp1-F7, F7-T3, T3-T5, T5-O1, Fz-Cz, Cz-Pz (see Fig. 1). Further details of the neonatal EEG recording method can be found in Tokariev *et al.* and Nevalainen *et al.*^23,24^.

### EEG Annotation

EEG files were de-identified and randomised before annotation. Each EEG file was annotated for seizures by three experts using the Nicolet Reader software (Natus, USA). Annotations were then exported into a text file using the start (onset) and duration of each seizure, considering all available derivations, with one second resolution. Annotations, therefore, did not include information on the location or nature of the seizure. Experts were requested to annotate seizures using the well-established definition: *a distinct, abnormal electrographic event with a clear beginning and end comprising sustained, repetitive evolving spike/sharp waves or rhythmic waveforms*^25^. This event was defined as a seizure if it was over 10 s in duration. Initial settings were a paper speed of 30 mm/sec (~12 s per screen), a sensitivity of 100 μV/cm with frequency cut-offs of 0.5 Hz (low) and 70 Hz (high), but experts were permitted to alter these settings for each recording to achieve the best possible performance.

Each expert had over 10 years of experience in the visual interpretation of neonatal EEG (LL, SV, JM)^20^. They were blinded to the clinical details of infants, and were only aware that the neonate had clinical suspicion of seizures. The annotations were based on the EEG signal. An electrocardiogram channel was also available for interpretation. Additional information such as video and other polygraphic channels were not available.

EEG recordings were performed as standard of care at the Helsinki University Hospital, Finland. Permission to publicly release the de-identified EEG recordings was received from the local ethics committee of the Children’s Hospital, Helsinki University Hospital, Finland.

### Code availability

Code has been uploaded to read the EDF files using Matlab (Mathworks, Natick, USA; read_data_montage.m to read the montage used for annotations; this function also calls read_edf.m). It is available at Github (https://github.com/ktapani/Neonatal_Seizure_Detection).

## Data Records

**Figure 2:**
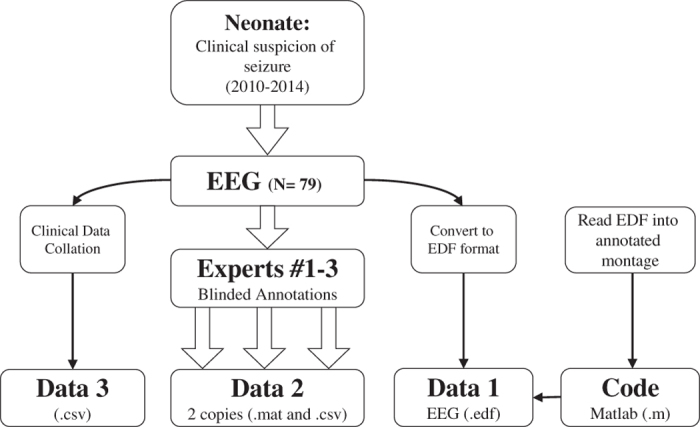
The structure of the database. Data 1, Data 2 and Data 3 can be found at Zenodo (Data Citation 1) Code can be found at https://github.com/ktapani/Neonatal_Seizure_Detection.

The dataset is available at Zenodo (Data Citation 1). A summary of the database structure is shown in Fig. 2. EEG files, annotations and clinical details are linked using a study ID/neonate number (1 to 79). EEG files were stored in a referential montage using an open source EDF format (http://www.edfplus.info/specs/edf.html).

**Figure 3:**
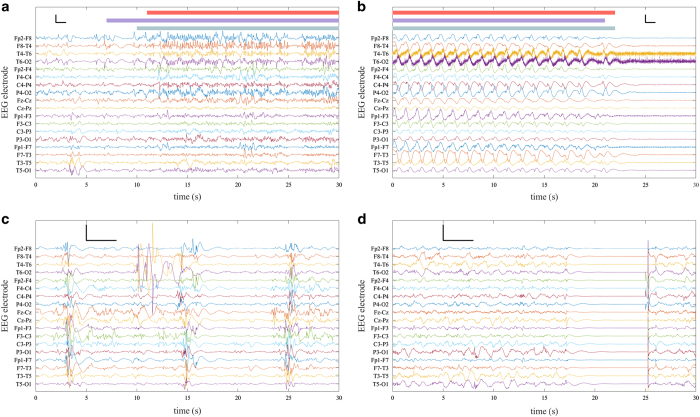
Example 30 s segments of EEG from the dataset. (**a**) The onset of a neonatal seizure discharge in a neonate with a right temporal haemorrhage (neonate 50; at 37 min 39 s). The seizure is prominent on the right side of the brain in derivations: Fp2-F8, F8-T4, T4-T6, T6-O2, and P4-O2. (**b**) The cessation of a widespread seizure discharge in a neonate with severe asphyxia (neonate 44; at 5 min 53 s); note, the muscle artefact in T6-O2 and T4-T6. (**c**) A period of burst suppression in a neonate with nonketotic hyperglycinemia interrupted by a high amplitude artefact at approximately 10 s on the T4-T6 and T6-O2 derivations (neonate 26; at 1 hr 2 min 34 s). (**d**) Recording pause expressed as zero EEG amplitude for approximately 8 s (neonate 42; at 38 min 6 s). The calibration bar (in black) denotes 100 μV (y-direction) and 1 s (x-direction) for (**a**) and (**b**), and 200 μV (y-direction) and 3 s (x-direction) for (**c**) and (**d**). In (**a**) and (**b**), annotations are plotted above the EEG signals (red, purple and green bars). Each bar denotes an independent, blinded annotation of the seizure by one expert. Data are filtered with a 6-pole Butterworth high-pass filter with a cut-off frequency of 0.5 Hz and an infinite impulse response, notch filter with a center at 50 Hz and bandwidth of 4/256 Hz.

Annotations were stored in a Matlab MAT format and CSV file format. The MAT file contains a cell array with 79 elements, where each element corresponds to a study ID number. Each element of the cell array is an M by N array, where M is the number of experts (M = 3), N is the duration of the annotation in seconds (variable) and each second is ascribed a 1 (denoting seizure) or 0 (denoting non-seizure). There are three CSV files (A, B, C), where each file contains the annotations of an expert. Within each CSV file, there are 79 columns where each column corresponds to a study ID number and each row is the annotation of one second of the EEG recording (1 for seizure and 0 for nonseizure). The EEG recordings and annotations files are synchronized in time (i.e. they start at the same time) and can be overlaid as shown in Fig. 3a,b.

Clinical data, extracted from patient reports, are presented in a speadsheet (CSV file format). Data include study ID/neonate number, gender, birth weight, gestational age (GA; in weeks), postnatal age of EEG recording in days, primary neurologically relevant diagnosis, as well as neuro-radiological findings (magnetic resonance imaging acquisition: Philips Intera Achieva 1.5 T and cranial ultrasound acquisition: GE LOGIQ S8). Several variables were defined categorically to aid de-identification.

The files in the dataset were defined as:

- **eeg (1 to 79):** EDF files containing the EEG recording using a referential montage from study ID 1-79 sampled at 256 Hz. EEG units are microvolts.
- **annotations_2017:** CSV file (A, B or C) or MAT file containing the annotations of 3 experts for all 79 neonates sampled every second (1 Hz).
- **clinical_information:** CSV file containing clinical data acquired from patient notes as well as information on the general spatial distribution of consensus seizures within the recording (marked only by reviewer A) aligned with EDF file.

## Technical Validation

Data were exported with all software filters of EEG acquisition turned off. The frequency response of the EEG signals will, therefore, only be affected by the bandwidth of the Nicolet v44 amplifier (defined at the lower frequency by the amplifier’s AC circuit and the higher frequency by the low-pass filter adjusted to the Nyquist rate).

**Figure 4:**
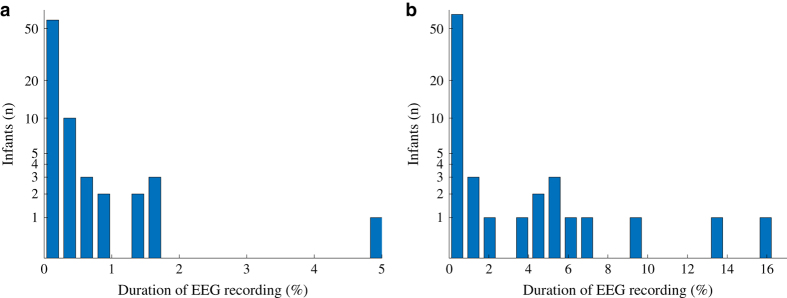
Distribution of periods within EEG recordings that are not cortical in origin. (**a**) High amplitude activity greater than 500 μV (see Fig. 3c). (**b**) Recording pauses (zero EEG activity; see Fig. 3d). Note, the y-axis has a logarithmic scale for clarity.

The electrode-skin interface was prepared by cleaning and rubbing the skin then applying a conductive gel. The process was repeated with a target impedance of less than 10 kΩ. A qualitative inspection of the EEG recording was performed by a skilled EEG technician to ensure the recorded activity was sufficient for accurate interpretation. EEG data includes technical artefacts that originate from recording pauses (resulting in periods of zero voltage; see Figs 3d and 4b), mains noise, as well as movement and muscle artefacts and other sources of excessive voltage (>500 microvolt; see Figs 3c and 4a). These artefacts are unavoidable and common during recordings in a clinical environment, especially in the recordings of critically ill neonates.

We used measures of EEG spectral power (averaged across all derivations and averaged differences between right and left hemispheres) to summarise the EEG recordings in the dataset. For a dataset of high quality EEG recordings, we would expect a high ratio in EEG power between low (cortical activity) and high (noise) frequency bands and equal power between left and right hemispheres.

The noise floor of the EEG recordings was measured using the power spectral density (Periodogram, with periods of excessively high artefact and recording pauses ignored), with average power in the 70–128 Hz band assumed to be indicative of noise. The noise floor was averaged across all 18 derivations in the bipolar montage and the median noise floor across EEG recordings in the dataset was 0.208 μV^2^/Hz (IQR: 0.041–0.498; n = 79). This can be compared to the average power in a lower frequency band (0.5 Hz–16 Hz; a band known to contain cortical activity) which has a median power of 202.75 μV^2^/Hz (IQR: 83.27–356.73; n = 79). Asymmetry in the recordings was measured using the revised brain symmetry index (rBSI^26^; a value of −1 implies bias towards the right, 0 implies symmetry, and 1 implies a bias towards the left). This measure compares the spectra from right hemisphere derivations to left hemisphere derivations and was calculated on a filtered version of the EEG data (50 Hz IIR notch filter and a 6-pole Butterworth high-pass filter with a cutoff at 0.5 Hz) inclusive of recording pauses and high amplitude artefacts. The median rBSI across EEG recordings was −0.001 (IQR: −0.207 to 0.161; n = 79).

Inter-observer agreement between experts was also estimated on the data using Fleiss’ kappa statistic^27^. Kappa was 0.767 (95%CI: 0.663–0.834). The prevalence and bias indices were 0.729 and 0.025, respectively. A summary of the annotations of each reviewer is given in Table 1. A total of 57 infants had a seizure annotated by at least one reviewer, 18 infants had a seizure annotated by at least 2 reviewers and 39 had a seizure annotated by all 3 reviewers. Out of 1379 seizures marked by all reviewers, 889 (65%) were annotated by all 3 reviewers (consensus), 295 (21%) were annotated by 2 reviewers (14%) and 195 were annotated by only a single reviewer.

## Usage Notes

We have successfully opened the EDF files using the following EEG viewers: EDF Browser (https://www.teuniz.net/edfbrowser/; version 1.66), Nicolet EDF Viewer/Reader (version 5.94.1.534), BESA (version 5.3) and Persyst (version 12). We have successfully imported EDF files into Matlab using our code available from Github, Fieldtrip (http://www.fieldtriptoolbox.org/; version 20190102) and EEGLab (https://sccn.ucsd.edu/eeglab/index.php; version 14.1.2b, with BIOSIG plugin). We have also successfully imported EDF files into Python using MNE (https://martinos.org/mne/stable/index.html; version 0.17.0) and Visbrain (http://visbrain.org/; version 0.4.4).

For researchers of automated algorithms of EEG analysis, additional material can be found on Github (https://github.com/ktapani/Neonatal_Seizure_Detection). These repositories contain code to open EDF files, feature extraction algorithms, several seizure detection algorithms, methods to evaluate algorithm outputs and a subset of per channel annotations of consensus seizures.

There are some common limits related to all datasets available from seizures in real patients. The amount of technical and biological artefacts present in clinical EEG data may challenge both visual and computational signal analysis (see Fig. 4). Many of them are unavoidable when recording in an electrically hostile environment such as the NICU where patients are connected to multiple other medical devices for life support. Artefacts of biological origin (movements, ECG, respiration) may also interfere as patients’ behaviour cannot be restricted. The present dataset includes recordings performed by technicians with more than ten years of specialist training in children’s EEG recordings; hence, this dataset represents the highest quality of EEG recordings available.

The overall seizure burden (proportion of EEG time covered by seizures) in any EEG recording is typically low. The ‘snap shot’ EEG recordings in this dataset were acquired during periods with a high likelihood of seizure and as such have a higher relative seizure burden compared to untargeted, long-term brain monitoring data^28^.

The number of recording channels has an impact on seizure capture. Most current long-term EEG monitoring uses 2 to 4 recording electrodes, and the international gold standard for neonatal EEG includes only 8–10 recording electrodes. We have collected neonatal EEG routinely with the international 10–20 system to improve the spatial resolution of cerebral events^29,30^. Our recent study showed that a higher number of electrodes leads to a recognition of more seizures, and this dataset provides a unique opportunity to compare the interactions between seizure location and various EEG montages^20^.

The experts used to annotate the EEG recordings for seizure are all from a single centre; although the level of agreement for these short recording is comparable to multi-centre comparisons for the annotation of neonatal EEG seizures^28^. While this limits the generalizability of the published annotations, researchers have the opportunity to overcome such a limit by incorporating their own annotation into future analyses; an advantage of public release of this dataset.

Neonatal seizures have variable aetiology (underlying cause, pathophysiology), and their EEG presentations vary widely with respect to waveforms, durations and spatial locations. This dataset was collected from a large neonatal unit (our collection periods represents approximately 80,000 births), and the selection in our cohort reflects the spectrum of neonatal seizure aetiologies and EEG waveforms. This is a strength of our dataset.

The gold standard of neonatal seizure detection remains visual detection in the EEG by the human expert. Recent studies have highlighted the relative ambiguity of experienced EEG experts^20,28^. Here, we provide annotations from three experts, which permits variation in study design from the use of a single expert’s annotation (as in most studies in the literature) to consensus annotations or other alternative combinations.

## Additional information

**How to cite this article**: Stevenson, N. J. A dataset of neonatal EEG recordings with seizure annotations. *Sci. Data*. 6:190039 https://doi.org/10.1038/sdata.2019.39 (2019).

**Publisher’s note**: Springer Nature remains neutral with regard to jurisdictional claims in published maps and institutional affiliations.

## Supplementary Material



## Figures and Tables

**Table 1:** A summary of the annotations of three reviewers.

	A (n=46)	B (n=45)	C (n=53)
Seizure Burden (mins)	10.2 (4.3–23.7)	15.0 (6.6–30.3)	8.6 (2.1–22.5)
Mean Seizure duration (s)	98 (48–246)	103 (67–288)	82 (38–175)
Seizures	5 (2–12)	6 (2–13)	6 (3–11)
Seizure burden is defined as the accumulated duration of seizure, in minutes, within a recording, mean seizure duration is the duration of seizure events, in seconds, within a recording and seizures is the number of seizure events in a recording. Results are presented as median (IQR), n is the number of infants with at least one seizure annotated by each reviewer.			
